# Deletions of multidrug resistance gene loci in breast cancer leads to the down-regulation of its expression and predict tumor response to neoadjuvant chemotherapy

**DOI:** 10.18632/oncotarget.6953

**Published:** 2016-01-20

**Authors:** Nikolai V. Litviakov, Nadezhda V. Cherdyntseva, Matvey M. Tsyganov, Elena M. Slonimskaya, Marina K. Ibragimova, Polina V. Kazantseva, Julia Kzhyshkowska, Eugeniy L. Choinzonov

**Affiliations:** ^1^ Laboratory of Oncovirology, Tomsk Cancer Research Institute, Tomsk, Russian Federation; ^2^ Laboratory of Translational Cell and Molecular Biomedicine, National Research Tomsk State University, Tomsk, Russian Federation; ^3^ Laboratory of Molecular Oncology and Immunology, Tomsk Cancer Research Institute, Tomsk, Russian Federation; ^4^ Department of General Oncology, Tomsk Cancer Research Institute, Tomsk, Russian Federation; ^5^ Department of Innate Immunity and Tolerance, Institute of Transfusion Medicine and Immunology, Medical Faculty Mannheim, University of Heidelberg, Mannheim, Germany; ^6^ Department of Head and Neck Cancer, Tomsk Cancer Research Institute, Tomsk, Russian Federation

**Keywords:** breast cancer, microarray analysis, chromosomal abnormalities, expression of multidrug resistance genes, neoadjuvant chemotherapy

## Abstract

Neoadjuvant chemotherapy (NAC) is intensively used for the treatment of primary breast cancer. In our previous studies, we reported that clinical tumor response to NAC is associated with the change of multidrug resistance (MDR) gene expression in tumors after chemotherapy. In this study we performed a combined analysis of MDR gene locus deletions in tumor DNA, MDR gene expression and clinical response to NAC in 73 BC patients. Copy number variations (CNVs) in biopsy specimens were tested using high-density microarray platform CytoScanTM HD Array (Affymetrix, USA). 75%–100% persons having deletions of MDR gene loci demonstrated the down-regulation of MDR gene expression. Expression of MDR genes was 2–8 times lower in patients with deletion than in patients having no deletion only in post-NAC tumors samples but not in tumor tissue before chemotherapy. All patients with deletions of ABCB1 ABCB 3 ABCC5 gene loci – 7q21.1, 6p21.32, 3q27 correspondingly, and most patients having deletions in ABCC1 (16p13.1), ABCC2 (10q24), ABCG1 (21q22.3), ABCG2 (4q22.1), responded favorably to NAC. The analysis of all CNVs, including both amplification and deletion showed that the frequency of 13q14.2 deletion was 85% among patients bearing tumor with the deletion at least in one MDR gene locus versus 9% in patients with no deletions. Differences in the frequency of 13q14.2 deletions between the two groups were statistically significant (*p* = 2.03 ×10^−11^, Fisher test, Bonferroni-adjusted *p* = 1.73 × 10^−8^). In conclusion, our study for the first time demonstrates that deletion MDR gene loci can be used as predictive marker for tumor response to NAC.

## INTRODUCTION

ATP-binding cassette (ABC) transporters are known to modulate the transport and metabolism of endogenous and exogenous substrates. They exercise protective physiological roles by removing potentially harmful molecules and can cause the efflux of common chemotherapeutic agents, which provide the mechanisms for multidrug resistance (MDR) to hormonal, targeted and cytostatic therapy in many cancer types, including breast cancer (BC) [1–4].

Neoadjuvant chemotherapy (NAC) is increasingly used for the treatment of primary breast cancer to downstage tumors, allows breast-conserving surgery [5] and improves breast cancer survival. In our previous studies, we reported that the clinical tumor response to NAC appeared to be associated with changes in the expression of the MDR gene in tumors after chemotherapy. We have shown that the favorable tumor response to NAC correlates with MDR gene down-regulation within breast tumors after NAC [6]. In addition, we have recently found that MDR gene down-regulation after NAC is associated with higher 5-year metastatic-free survival rates in breast cancer patients. In contrast, poor clinical response to NAC appeared to be related to the increase in MDR gene expression in tumor tissue after NAC and poor disease prognosis [7].

Because the physiological function of ABC transporters is the efflux of xenobiotics, including chemotherapeutic drugs [1], the increased expression of MDR genes is the expected cell response to chemotherapy. This phenomenon is commonly known and has been shown to be associated with drug-resistance in many *in vitro* studies. Accumulating evidence indicates that MDR gene up-regulation is induced by several genetic and epigenetic mechanisms, such as DNA hypomethylation, histone deacetylation, activation of available transcription factors and miRNA expression, as well as the functioning of MDR-related signaling pathways [8–15]. However, relatively little is known about MDR down-regulation in clinical breast cancers and only a few *in vitro* studies are available. New ABC down-regulation mechanism has been shown in SGC7901/VCR gastric cancer multi-drug resistant cell line. Such ABC transporters as ABCB1, ABCC5 and ABCG1 were found to be direct targets of miR-129–5p. Wu Q et al. demonstrated that miR-129–5p over-expression resulted in the reduced chemo-resistance of SGC7901/VCR and SGC7901/ADR cells [16]. Salvamoser J.D., et al. revealed a novel mechanism of BCRP down-regulation in human brain capillaries. The *N*-methyl-d-aspartate (NMDA) receptor was shown to be involved in the down-regulation of BCRP, that was confirmed by examining BCRP transport function and expression after exposure to NMDA [17].

In clinical settings in addition to our research data, Kim B. et al. have reported the reduction in protein expression of P-glycoprotein and Bcrp in breast tumors after NAC compared with that obtained in biopsies taken before treatment in 26% and 41% of patients, respectively. However, the mechanisms that contributed to MDR gene down-regulation after chemotherapy was not discussed [18]. Demidenko R., et al. found that eight ABC transporter genes (*ABCA8*, *ABCB1, ABCC6*, *ABCC9*, *ABCC10*, *ABCD2*, *ABCG2*, and *ABCG4*) displayed markedly down-regulated expression in prostate cancer in comparison with nonmalignant prostate tissues, that was associated with gene promoter methylation [19].

Therefore, decreased MDR gene expression in breast tumors after neoadjuvant chemotherapy is associated with its effectiveness, but the molecular mechanism for MDR down-regulation is poorly understood. We did not find any correlation between clinicopathological features and negative regulation of MDR gene expression in breast tumors during therapy [6].

Further study is needed to clarify the mechanisms responsible for MDR gene down regulation in breast cancer patients treated with NAC, which could identify novel predictive markers for the response to NAC and novel targets for regulating the MDR phenotype.

We hypothesized that the negative regulation of MDR gene expression in tumors of patients who received chemotherapy was associated with deletions of MDR gene loci. It is well known that DNA copy number variations (CNVs), such as deletions and amplifications, are major genomic alterations in breast and other tumor sites (www.progenetix.org [20]) that contribute to tumor progression and response to chemotherapy. Furthermore, deletions of DNA loci are known to result in the reduced expression of genes located in these regions [21, 22]. In contrast, amplification of gene loci containing ABC transporters was shown to be associated with poor prognosis. Germinal center B-cell-like (GCB) subtype of diffuse large B-cell lymphoma (DLBCL) was characterized by more gains at 7q22.1, which contained ABC transporter ABCB1 compared to activated B-cell-like (ABC) lymphoma, according to microarray analysis [23]. Dunleavy K et al. demonstrated a significantly higher response (83% vs 13%; *p* < 0.001) and median overall survival (10.8 vs 3.4 months; *p* = 0.003) in ABC compared with GCB DLBCL, treated with doxorubicin and bortesamib [24].

Kim I.-W., et al. reported that one hundred and fifty-two pharmacogenes were tested for CNV frequencies in several tumors (hepatocellular carcinoma, lung squamous cell carcinoma, acute myeloid leukemia, and lymphoid neoplasm diffuse large B-cell lymphoma) using The Cancer Genome Atlas dataset [25]. Authors suppose that germ line and somatic CNVs of genes involved in drug metabolism and efflux may contribute to patient's variations in drug responses and serve as promising biomarkers to increase the benefits in cancer treatment [25].

In our study, we performed a combined analysis of deletions of MDR gene loci in tumor DNA, MDR gene expression and clinical response to neoadjuvant chemotherapy in BC patient cohorts. We designed an integrated, analytical method to identify the chromosome regions in which CNVs were correlated with MDR gene down-regulation and the response to NAC by taking genomic data on RNA gene expression and variations in DNA copy number from the same group of patients.

## RESULTS

**Figure 1:**
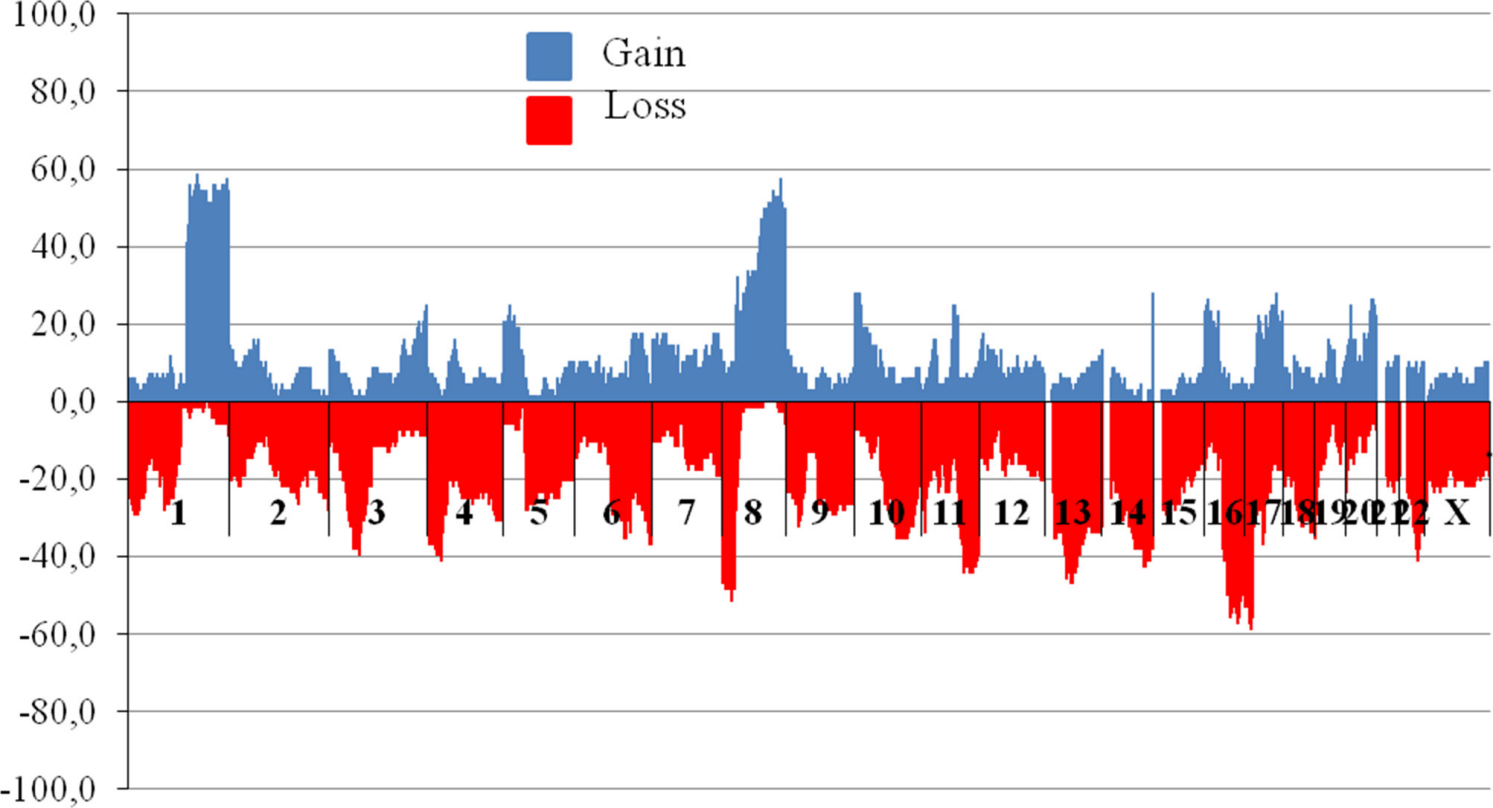
The frequency of CNVs in breast tumor DNA before treatment Abscissa – cytobands (*n* = 852), axis of ordinates – frequency of patients bearing CNV in tumor (%). Blue (upper horizontal dotted) and red (under horizontal dotted) are regions of gains (amplifications) and losses (deletions), respectively. For CNV study microarray analysis was performed using high density microarray platform Affymetrix (USA) CytoScan™ HD Array.

Structural CNVs have been observed in various chromosomal regions in breast tumor. Figure 1 demonstrates the frequency of copy number variations including deletions and amplifications in cytobands of all BC patients. It is important that only in two chromosomes, namely chromosome 1 and 8, tumor DNA amplifications have been found in more than 30% of patients. The frequency of DNA amplification in chromosome 1 long arm (1q21.1 – 1q44 regions) was detected in the range from 41% to 59% of the total tumor sample number.

Amplifications are observed in short (8p11.22 in 32.4% of cases) and long arms of chromosome 8 (8q12.1 – 8q24.3; from 32% to 57% of cases). In 8q11.21 – 23, 10p15.3 – 14, 14q32.33, 16p13.13, 17q24.2, 20q13.2 *u* 20q13.21 chromosomal regions amplifications were detected in 25–30% of patients. Less frequently observed amplifications with the frequency range from 25% to 30% of cases occur in regions 8q11.21 – 23, 10p15.3 – 14, 14q32.33, 16p13.13, 17q24.2, 20q13.2 and 20q13.21.

Deletions with a frequency of more than 50% were detected in chromosomal regions: 8p21.2, 16q21 – 24.1 and 17p13.3 – 17p11.2. Deletion of regions: 4p14, 8p23.3 – 21.1, 11q22.1 – 24.3, 13q14.11 – 14.3, 13q14.2 – 14.3, 14q32.11 – 32.31, 16q12.1 – 13, 16q24. 2 – 24.3 and 22q13.1 occurred in 40–50% of cases. In general, the spectrum of chromosomal regions bearing CNV, includes areas 1q, 8p, 8q, 11q, 16q, 17p and our results are consistent with published data [26]. Additionally, CNV have been found in 13q and 14q32 regions.

A large number of numerical CNV, which is mainly represented by a single chromosome monosomy is also observed in breast tumor. Aneuploidy occurs in more than half of the samples, trisomies are observed much rarer. Monosomy of chromosome 13 was the most common (19 out of 68 patients – 28%) followed by monosomy of chromosome 14 (12/68 – 18%), chromosome 15 (9/68 – 13%), chromosomes 4 and 18 (7/68 each – 10%), chromosome 17 (6/68 - 9%), 9 and X chromosome (5 cases each – 7%).

Monosomies were not seen in only 3 chromosomes: 1, 8 and 20, thus indicating their important role in maintaining the viability of tumor cells. This is confirmed by the presence of frequent structural CNV as amplifications mentioned above. The frequency of trisomy in some chromosomes was not exceed 4% (3 of 68 cases) and trisomies of chromosomes 7, 8, 12, 17, 20 and 21 were noted in 2–3 cases.

### MDR gene locus deletions associated with gene expression and tumor response to chemotherapy

In the present study, we analyzed the chromosomal deletions of loci containing the MDR gene in pre-NAC tumor samples in relation to the corresponding MDR gene expression and response to neoadjuvant chemotherapy. MDR gene expression profiling and detection of tumor-associated copy number variations in DNA of the same tumors were performed. The list of investigated MDR genes included the main ABC family transporters ABCB1, ABCB3, ABCC1, ABCC2, ABCC5, *ABCG1*, *ABCG2* and *MVP*. The corresponding MDR gene loci that were deleted in the tumors are also listed in Table 1.

**Table 1:** The frequency of MDR gene locus deletion in breast tumors in relation to down-regulation of MDR gene expression and clinical response to NAC

Genes	Gene-loci	Number of cases with the down-regulation in MDR gene expression after NAC	The frequency of cases with the decrease in MDR gene expression in patients with deletion of MDR loci, %	Total number of MDR loci deletion	Frequency of MDR locus deletion in cases with decreased MDR gene expression after NAC	The frequency of clinical response to NAC in patients with deletion of MDR loci, %
*ABCB1*	7q21.1	39	100	10	10 (25.6%)	100
*ABCB3*	6p21.32	41	86	7	6 (14.6%)	100
*ABCC1*	16p13.1	34	88	8	7 (20.6%)	88
*ABCC2*	10q24	41	83	24	20 (48.8%)	71
*ABCC5*	3q27	33	75	4	3 (9.1%)	100
*ABCG1*	21q22.3	41	77	13	10 (24.4%)	62
*ABCG2*	4q22.1	37	79	14	11 (29.7%)	79
*MVP*	16p11.2	40	86	7	6 (15.0%)	86

The clinical response to NAC – complete response + partial response; for study the deletions of MDR gene loci, microarray analysis was performed using high density microarray platform Affymetrix (USA) CytoScan^™^ HD Array.

Among the patients who had tumors with deletions of MDR gene loci, 75%–100% persons appeared to display down-regulation of the expression of the same MDR gene (the decrease in gene expression in post- NAC tumor samples compared to the matched pre-NAC specimens). It should be noted that only in 9%–49% of patients who showed down-regulation of MDR gene expression during the NAC had loci deletions (Table 1). All patients (100%) with deletions of the ABCB1, ABCB3 and *ABCC5* gene loci, 7q21.1, 6p21.32 and 3q27, respectively, were found to respond favorably to NAC. The clinical response to NAC in BC patients showing ABCC1 (16p13.1), ABCC 2 (10q24),*ABCG1* (21q22.3) and *ABCG2* (4q22.1) gene loci deletions varied little, ranging from 62% to 88%.

The frequency of tumors with one or more loci containing ABC family genes equals to 61% (14/23) in CAX treated patients, 56% (20/36) in FAC regimen and 36% (5/14) in taxotere based regimen (*p* > 0.05 Fisher test). The frequency of clinical response to NAC was appeared to correlate with the number of deleted loci containing ABC genes. Tumor response was detected in 83–100% patients who had deletions in 2–5 ABC genes (Table 2). No difference in the frequency of response to NAC between tumors having no deletions and tumors with deletion in only one ABC transporter gene was found.

**Table 2:** The frequency of clinical response to NAC in breast cancer patients in relation to ABC gene loci with deletions in pre-NAC tumor samples

Numbers of deletions of ABC transporters gene loci	Number of patients	The frequency of clinical response to NAC
0/8	34	17/34 (50.0%)
1/8	14	7/15 (46.7%)
2/8	6	5/6 (83.3%)
3/8	7	6/7 (85.7%)
4/8	8	7/8 (87.5%)
5/8	3	3/3 (100%)

The clinical response to NAC – complete response + partial response.

These data confirm good associations between MDR loci deletions and NAC efficiency.

### MDR gene deletions affect gene expression in post-NAC tumor samples

The next step was to assess whether the existence of a deletion in the chromosome region in which MDR genes were located affected their expression in tumors before and after NAC. We examined the MDR gene expression level in pre-NAC and post-NAC tumor samples in patients who had MDR gene deletions versus patients without deletions(Table 3). We did not find any differences in the expressions of examined gene between the tumors with and without deletions, collected before NAC. However, measurement of the MDR gene expression in post-NAC samples showed that the expression level was 2.5 to 7.5 times lower in tumors lacking the MDR gene loci than in tumors without the deletion. Significant differences were revealed for the expression levels of *ABCB1*, *ABCC1*, *ABCC2*, *ABCG1* and *ABCG2* genes (Table 3).

**Table 3:** MDR gene expression level in pre-NAC and post-NAC samples of breast tumor with or without MDR gene loci deletions

Genes	Sighting point	Tumor with no deletion	Tumor with deletion	*p*-value
*ABCB1*	Pre-NACT	3.18 ± 0.73	4.05 ± 1.81	0.584
	Post-NACT	**4.29** ± **1.25**	**0.57** ± **0.28**	**0.028**
*ABCB3*	Pre-NACT	0.85 ± 0.09	1.15 ± 0.69	0.416
	Post-NACT	0.90 ± 0.22	0.46 ± 0.18	0.253
*ABCC1*	Pre-NACT	1.33 ± 0, 32	0.83 ± 0.25	0.689
	Post-NACT	**1.53** ± **0.37**	**0.43** ± **0.16**	**0.047**
*ABCC2*	Pre-NACT	3.54 ± 0.91	2.22 ± 0.72	0.096
	Post-NACT	**3.77** ± **1.36**	**1.55** ± **0.59**	**0.032**
*ABCC5*	Pre-NACT	2.28 ± 0.43	3.34 ± 1.33	0.194
	Post-NACT	2.70 ± 0.45	3.08 ± 1.35	0.560
*ABCG1*	Pre-NACT	2.03 ± 0.47	1.12 ± 0.44	0.409
	Post-NACT	**1.78** ± **0.39**	**0.33** ± **0.10**	**0.009**
*ABCG2*	Pre-NACT	2.02 ± 0.38	1.93 ± 0.88	0.306
	Post-NACT	**2.50** ± **0.51**	**0.78** ± **0.34**	**0.044**
*MVP*	Pre-NACT	0.51 ± 0.12	0.46 ± 0.21	0.990
	Post-NACT	0.60 ± 0.16	0.21 ± 0.08	0.275

*p*-value – Mann-Whitney *U*-test was applied to identify the link between the expression levels of MDR genes in breast tumors and the presence/absence of deletion in the MDR loci. The expression levels of the MDR genes were measured using real-time quantitative PCR (RT-qPCR) based on the TaqMan technology. Expression levels are shown as mean and standard error (M ± SE).

### MDR gene locus deletions associated with lack of 13q14.2 region

**Figure 2:**
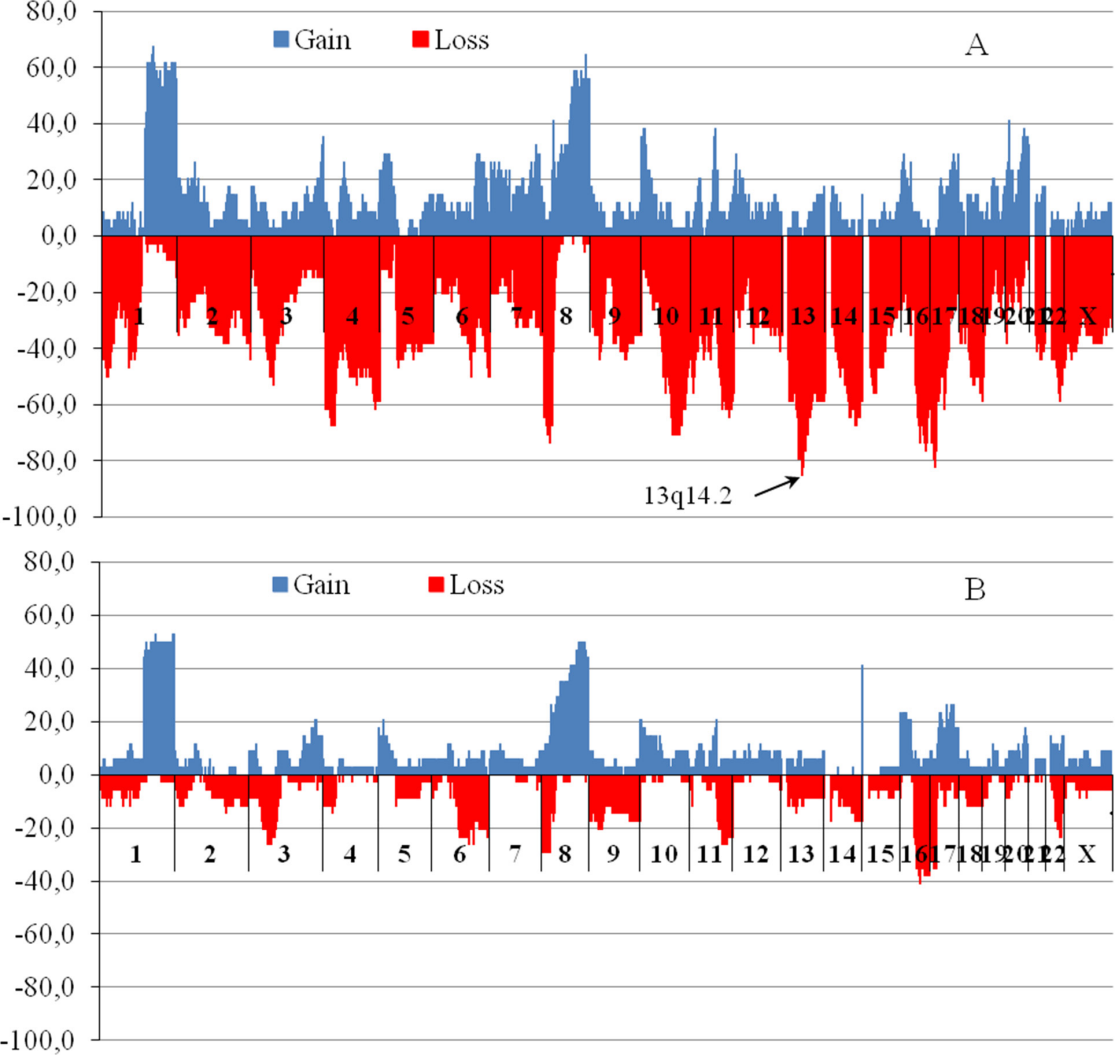
The chromosomal regions with copy number variations for patients with MDR gene locus deletions (A) and patients without deletions (**B**). Blue (upper horizontal dotted) and red (under horizontal dotted) are regions of gains (amplifications) and losses (deletions), respectively. Abscissa – cytobands (*n* = 852), axis of ordinates – frequency of patients bearing CNV in tumor (%). For CNV study microarray analysis was performed using high density microarray platform Affymetrix (USA) CytoScan™ HD Array.

The next step was to examine the CNV in the cytogenetic bands that most often occurred in tumors with MDR loci deletions. All patients were divided into two groups: the first group consisted of patients who had at least one of the observed MDR gene locus deletions (*n* = 34), the second group included patients who did not have deletions in MDR gene loci (*n* = 34). Figure 2 shows the frequency of patients with all CNVs that were detected in all 852 chromosomal cytobands among the patients with a deletion in the MDR gene loci (Figure 2A) and those without deletions (Figure 2B). The analysis of all CNVs, including amplifications and deletions showed that the frequency of the 13q14.2 deletion was 85% (29/34) among patients bearing a tumor with a deletion at least in one MDR gene locus. In patients who had no deletions of MDR gene loci, 13q14.2 loss was detected in only 9% (3/34) of cases. Differences in the frequency of 13q14.2 deletions between the two groups were statistically significant (*p* = 2.03 × 10^−11^, Fisher test, Bonferroni-adjusted *p* = 1.73 × 10^−8^).

Thus, our findings show that MDR gene deletion appears to down-regulate the expression of the corresponding gene in 75–100% of patients and provide good clinical response to NAC in 62–100% of cases. However, the reduction in MDR gene expression within breast tumors after NAC is not always associated with deletion of MDR gene loci. Expression of the MDR gene was found to be lower in patients with a deletion than in those with no deletion only in post-NAC tumor samples. No effect of a deletion on the level of MDR gene expression was revealed in tumor samples of patients before chemotherapy. Deletion of chromosomal region 13q14.2 occurred in 85% patients lacking the MDR gene within a tumor.

## DISCUSSION

In accordance with numerous data the increased expression of MDR genes is the expected response to chemotherapy [27–30]. In our previous study we have also demonstrated NAC induced up-regulated expression of MDR genes in breast cancer patients. We have shown the correlation between up-regulation of MDR gene expression and resistance to neoadjuvant chemotherapy. In contrast, in the group with tumor responses, we found a statistically significant down-regulation of expression of *ABCB1*, *ABCC1*, *ABCC2*, *ABCC5*, *ABCG1*, *ABCG2* and MVP genes following chemotherapy in breast cancer patients [6], and a decrease in MDR protein expression was revealed by Kim et al. (2013) [18]. Results of this study suggested that deletion of the MDR gene locus in tumors might be one of the causes of down-regulation of MDR gene expression during chemotherapy.

The obtained data indicated that tumors with a deletion of at least one gene locus are sensitive to NAC. A good NAC clinical response rate in patients with MDR locus deletion ranged from 62% to 100% for different genes, thus reflecting the close correlation between MDR gene locus deletions and good response to NAC. This finding is consistent with the data of Flahaut et al., (2006), who showed that the neuroblastoma cell lines IGRN-91 and LAN-1R with a deletion of the 7q region demonstrated a good response to doxorubicin chemotherapy [31]. However, only a few studies have investigated the loss of the MDR gene in relation to the corresponding gene expression and chemosensitivity of tumors. In contrast, the amplification of gene loci in the context of multidrug resistance has been more widely discussed. It is shown that the amplification of MDR gene loci endows tumor cell lines with chemoresistance, and elevated levels of MDR gene expression after repeated chemotherapy courses have been determined [31–34].

To understand whether deletion of MDR gene-containing chromosomal loci in tumors could be related to initial or chemotherapy-induced (acquired) drug resistance, we examined the expression of these genes before NAC and after chemotherapy. Because the expression level of the studied MDR genes in tumor samples collected before chemotherapy did not depend on the presence of MDR gene loci deletion (see Table 3), it may be proposed that haplotype insufficiency of the MDR gene locus in tumor cells cannot manifest itself before chemotherapy. However, under the influence of chemotherapeutic agents that activate ABC transporter efflux, we observed decreased MDR gene expression levels in tumors with a MDR gene deletion (see Table 3). Taking into account that the most well known mechanisms of MDR gene expression regulation aimed at the activation of their expression in response to chemotherapy, the question as to how the gene allelic deletion leads to down-regulation of MDR expression after NAC remains unclear. Several mechanisms that would allow this MDR gene expression decrease can be assumed. The first could be provided by allelic deletions resulting in a deficit of an ABC gene and subsequent protein expression in tumor cells in response to chemotherapy. Low levels of ABC transporters cannot sufficiently eliminate drugs, allowing tumor cells to become sensitive to chemotherapy and die. Tumor cell number reduction due to cell death also contributes to decreasing the total expression of the MDR gene.

The second mechanism could be related to various signaling pathways and transcription factors that are known to influence the response to ABC family genes mediated by MDR, including the Ras-mediated pathway, cyclic adenosine monophosphate (cAMP)/protein kinase A (PKA) pathway, phosphatidylinositol 3-kinase (PI3K)/protein kinase B (Akt) pathway, Y-box binding protein 1 (YB-1), phosphatase and tensin homologue (PTEN), p53, protein kinase C and other protein kinases [35]. When these pathways are deregulated, no gene expression activation will be provided in response to chemotherapy.

Chemotherapy is known to induce the cellular stress response. Leung and Sharp (2010) reported an interesting mechanism for the regulation of gene expression by microRNAs within stress-related conditions [36]. In the cells, a basal level of target mRNA expression and, simultaneously, a certain constant level of microRNAs, which down-regulate the target mRNA, is maintained, endowing their expression to be kept at a constant level. A level of target mRNA (e.g., the MDR mRNA) is dramatically raised following a stress response, saturating the threshold microRNA that results in its insufficiency to disrupt the mRNA and allows its translation. MDR gene haplotype insufficiency leads to a slow increase in the mRNA level in response to chemotherapy and does not have sufficient time to saturate the threshold microRNAs. As a result of deleted MDR genes, their expression in tumor cells is not sufficiently increased in response to treatment, and the tumor becomes chemosensitive.

Our data showed a high frequency (85%) of deletion in the 13q14.2 region in tumors, with the loss of at least of one MDR gene locus (see Figure 1). These data indicate that genes located in the 13q14.2 locus could regulate MDR gene expression when the MDR allelic deletion occurs. Therefore, loss of the 13q14.2 locus can be a marker of the MDR gene deletion in tumors. Because deletion of the 13q14.2 locus can be easily detected by PCR, this marker is potentially available for clinical use to predict chemotherapy response in breast cancer patients. The 13q14.2 locus is a region of chromosome 13 in which 2 gene loci, 28 protein-coding genes, 23 pseudogenes and 19 RNA genes are localized (http://www.genecards.org/). The tumor suppressor retinoblastoma1 (*RB1*) gene is the most famous among the protein-coding genes, and our results indicate that *RB1* haplotype insufficiency occurs in 85% of patients with a deletion of MDR gene loci. *RB1* loss is thought to function as a driver of mutations in this group, and this has been experimentally confirmed by Jiang, Jones et al. (2011) [37].

*RB1* has been shown to inhibit the E2F1 and E2F2 transcriptional factors [38]; therefore, the allelic deletion of *RB1* that was observed in 85% of breast cancer patients in our study is likely to lead to the activation of E2F1 and E2F2. However, evidence suggests that the *ABCB1* gene promoter does not contain E2F1 and E2F2 binding sites [39]. Moreover, E2F1 and/or E2F2 gene deletions were detected in 18 of 34 (52%) patients who lacked the 13q14.2 locus, which should decrease their expression.

In addition, a recent report shows that Histone Deacetylase 1 and 2 (*HDAC1* and *2*) have a reciprocal relationship to *RB1* and are able to reduce *ABCB1* and *ABCC2* gene and protein expression in colorectal adenocarcinoma and carcinoma cell lines [40]. pRb has been reported to recruit chromatin-modifying enzymes, such as *HDAC1*, through LXCXE motif-binding E2F transcription factors and inhibit *HDAC1* [38]. It can be assumed that haplotype deficiency of the *RB1* gene in tumors of patients with a MDR gene deletion results in increased activity of *HDAC1* and *2*, which, in turn, reduces the expression of the MDR genes. Further study is needed to understand why *RB1* gene deletions are associated with the loss of MDR gene loci.

The present study is believed to be the first to investigate the association between MDR gene deletions, down-regulation of the expression of the same gene and good clinical response to NAC. A close relationship between the loss of chromosomal loci containing the MDR gene and tumor response to chemotherapy shows the feasibility of using the MDR gene loci deletion as a prognostic assay to predict tumor response to NAC.

## MATERIALS AND METHODS

### Patients, tumors and treatments

Breast cancer patients (*n =* 73) with clinical stage IIA to IIIC (*T*_1–4_*N*_0–3_*M*_0_), in the age range between 26 and 69 years (median age 52.1 ± 0.46) were treated at the Cancer Research Institute (Tomsk, Russia) between 2006 and 2012 (Table 4).

**Table 4:** The clinicopathological parameters of BC patients, *n* = 73

Clinicopathological parameter		*N* (%)
Age (year)	≤ 45	23 (31.5)
	> 45	50 (68.5)
Menstrual status	Premenopausal	39 (53.4)
	Postmenopausal	34 (46.6)
Histological type	Invasive ductal carcinoma	63 (86.3)
	Invasive lobular carcinoma	3 (4.1)
	Medullary carcinoma	2 (2.7)
	Others	5 (6.8)
Tumor size	T_1_	9 (12.3)
	T_2_	57 (78.1)
	T_3_	3 (4.1)
	T_4_	4 (5.5)
Lymph node status	N_0_	30 (41.1)
	N_1_	33 (45.2)
	N_2_	4 (5.5)
	N_3_	6 (8.2)
Estrogen receptor	Positive	36 (49.3)
	Negative	33 (45.2)
	No data	4 (5.5)
Progesterone receptor	Positive	38 (52.1)
	Negative	31 (42.5)
	No data	4 (5.5)
HER2	0/+	51 (69.9)
	++	11 (15.1)
	+++	6 (8.2)
	No data	5 (6.8)
Molecular subtype	Luminal B	45 (61.6)
	Triple-negative	18 (24.7)
	HER2-positive	10 (13.7)
Histological form	Unicentric	50 (68.5)
	Multicentric	23 (31.5)
Pathomorphosis	1 rate	21 (28.8)
	2 rate	23 (31.5)
	3 rate	6 (8.2)
	4 rate	4 (5.5)
	No data	19 (26.0)
Grade	1 rate	2 (2.7)
	2 rate	53 (72.6)
	3 rate	6 (8.2)
	No data	12 (16.4)
NAC regimen	CAX	23 (31.5)
	FAC	36 (49.3)
	Taxotere	14 (19.2)
NAC response	Complete response	5 (6.8)
	Partial response	40 (54.8)
	Stable disease	20 (27.4)
	Progressive disease	8 (11.0)

Abbreviations: NAC, neoadjuvant chemotherapy; CAX, Cyclophosphamide-Adriamycin-Xeloda; FAC, 5-Fluorourail-Adriamycin-Cyclophosphamide; HER2 testing is performed in accordance with American Society of Clinical Oncology/College of American Pathologists Guideline 2007 Recommendation [41].

The procedures followed in this study were made in accordance with the Helsinki Declaration (1964, amended in 1975 and 1983). This study was approved by the institutional review board, and all patients signed an informed consent for voluntary participation.

All patients received two to four cycles of systemic neoadjuvant chemotherapy with the FAC (5-Fluorourail, Adriamycin, and Cyclophosphamide) or CAX (Cyclophosphamide, Adriamycin, Xeloda) regimens or Taxotere. Physical examination was performed before NAC and was repeated after two cycles of NAC and before surgery to determine the clinical response. Imaging of the primary breast lesion was performed with mammography and/or ultrasonography, and clinical and imaging responses were categorized according to the International Union Against Cancer criteria [42]. In this way, patients were grouped into clinical responders (CR and PR) and non-responders (SD and PD). Surgery (radical resection, sectoral resection or mastectomy) was performed within three to four weeks after the last administration of chemotherapy in responsive patients. After surgery, adjuvant chemotherapy or hormonal therapy was given, and fresh breast cancer tissues were obtained during the initial diagnostic biopsy (∼10 mm^3^) before NAC and in the course of tumor resection after NAC (∼60–70 mm^3^). The obtained tissue samples were stored in RNALater solution (Ambion, USA #AM- 7020) within 24 hours at +4°C and then at −80°C per the manufacturer's instructions until further use. Histological diagnosis was confirmed for all samples.

### RNA isolation and cDNA synthesis

Total RNA was extracted from 68 samples of pre- and post-NAC tumor tissues using RNeasy Plus Mini Kit DNase I digestion (Qiagen, Germany #74134), and RiboLock RNase inhibitor (Fermentas, Lithuania #EO0382) was added to the isolated RNA. Five patients showed a complete response, rendering it impossible to obtain further tumor samples. The RNA integrity number (RIN) was measured using the 2200 TapeStation Instrument and R6K ScreenTape (Agilent Technologies, USA #5067–5367). RNA with an RIN > 7 was reverse transcribed to cDNA using the RevertAid Kit with random hexanucleotide primers (Fermentas, Thermo Scientific #K1691) following the manufacturer's instructions.

### Expression analysis

The MDR gene expression was assayed in pre- NAC and post-NAC tumor samples (pre-treatment expression and post-treatment expression). The expression levels of the MDR genes were measured using real-time quantitative PCR (RT-qPCR) based on the TaqMan technology with a Rotor-Gene-6000 instrument (Corbett Research, Australia) as described in detail in [6]. The primer and probe sequences of *ABCB1*, *ABCC1*, *ABCC2*, *ABCC5*, *ABCG2*, *MVP* and *GAPDH* were given in our previous study [6]; and the primer and probe sequences of *ABCB2* and *ABCG1* were obtained from a previous paper [43]. One internal gene, *GAPDH*, was used to normalize the expression levels of the studied genes. The average C_t_ (cycle threshold) was estimated for the gene of interest and *GAPDH*, the relative expression was evaluated using the Pfaffl method [44], and a formula was used to determine the expression ratio between the sample and the calibrator [6]. The relative expression level was also normalized to a calibrator consisting of a pool of normal breast tissue specimens. For this purpose, adjacent normal breast tissue specimens from 10 breast cancer patients (NAC-free) were used as a source of normal RNA. The results were presented as n-fold differences in MDR gene expression relative to *GAPDH* and normal breast tissue.

### DNA isolation

DNA was extracted from 68 samples of pre-NAC tumor tissues using the QIAamp DNA mini Kit (Qiagen, Germany #51304), and the DNA concentration and purity were assessed using a NanoDrop-2000 spectrophotometer (Thermo Scientific, USA). The concentration level ranged from 50 to 150 ng/μl; the A_260_/A_280_ = 2.10–2.35; and the A_260_/A_230_ = 2.15–2.40. The integrity was evaluated by capillary electrophoresis using the 2200 TapeStation Instrument and Agilent Genomic DNA ScreenTape System Quick Guide (Agilent Technologies, USA # 5067–5365). The DNA mass was greater than 48 kbp.

### Microarray analysis

To study CNVs of MDR gene loci, microarray analysis was performed using high density microarray platform Affymetrix (USA) CytoScan^™^ D Array, (http://www.affymetrix.com/esearch/search.jsp?pd=prod520004&N=4294967292). The array contained 2.67 million markers, 1.9 million non-polymorphic markers and more than 750 000 single nucleotide polymorphism (SNP) markers that allowed structural variations of more than 36 000 genes to be determined. The presence of SNP markers on the microarray enabled copy number analysis to detect gains and losses in the DNA and loss of heterozygosity. Procedures of sample preparation, hybridization and scanning were performed in accordance with the manufacturer's protocol using the system Affymetrix GeneChip^®^ Scanner 3000 7G (Affymetrix, USA). The Chromosome Analysis Suite 2.0 software (Affymetrix, USA), which is specifically devised for analyzing microarray results from the CytoScan^™^ HD Array, was used. Unbalanced chromosomal aberrations (deletions and amplifications, or Loss and Gain) were detected in chromosomal regions 3q27.1, 4q22.1, 6p21.3, 7q21.12, 10q24.2, 16p13.11, 16p11.2 and 21q22.3. Because stromal elements and other normal cells were presented in tumor tissue samples, the percentage of normal genomic DNA was high in the obtained DNA. The Microchip CytoScan^™^ HD Array can detect at least 5% of mutant DNA. In nearly all cases, CNV were mosaic, i.e., mutant tumor DNA was detected along with the normal DNA. Additionally, the percentage of mutant DNA copy number state (CN-state) ranged from 15 to 88%. The Chromosome Analysis Suite 2.0 software graphically presents mosaicism as allele peaks with 4 bands (AAA, AAB, ABB, BBB). The snpQC value ranged from 13 to 25 and negatively correlated with the CNV frequency. Even if the snpQC values were low, the width of the allele peaks was “good” (according to the progenetix resource [45]) for CNV identification.

### Real-time PCR

Specific target sequences were selected for real-time quantitative PCR (qPCR) using the VectorNTI *11.5 software* (Life Technologies, USA). Two primers and probe were created for each lost chromosomal region. The sequence of the primers is shown in Table 5. We used *CASR* (calcium-sensing receptor) as a reference gene because it is localized in 3q13.33 region with low frequency of CNVs that points the reduced risk of DNA loss and gain (see Figure 1).

**Table 5:** Sequence of primers and probes used in the study

Loci	Genes	Primers	Sequence	Amplicon
7q21.12	*ABCB1* NG_011513.1	Forward	5′-ttcaggtcggaatggatctt-3′	118 bp
		Reverse	5′-gcaactatgtaaactatgaaaatgaaa-3′	
		Probe	FAM 5′-accgcaatggaggagcaaagaa-3′BHQ1	
6p21.32	*ABCB3 (TAP2)* NG_009793.3	Forward	5′-tactaacttgcctgggtcacata-3′	92 bp
		Reverse	5′-gtcagggagtataggcaactctt-3′	
		Probe	FAM 5′-agaggtggacttgcccagctttg-3′BHQ1	
16p13.1	*ABCC1* NG_028268.1	Forward	5′-tctctctggaattactgcgga-3′	101 bp
		Reverse	5′-acaggcatggagtcagctcta-3′	
		Probe	FAM 5′-ccccaagagctgtaagccaagtc-3′BHQ1	
10q24	*ABCC2* NG_011798.1	Forward	5′-gccacaggtatgtaagaaggatt-3′	95 bp
		Reverse	5′-tggatactgagcagttcaggaa-3′	
		Probe	FAM 5′-catgggtggaatggtaaatcaatatc-3′BHQ1	
3q27	*ABCC5* NC_000003.12	Forward	5′-gtcccaaccaaatcagaggt-3′	108 bp
		Reverse	5′-cctggtgctatattgtcaagaca-3′	
		Probe	FAM 5′-cctcagaagcacccatgttagaaca-3′BHQ1	
21q22.3	ABCG1 NC_000021.9	Forward	5′-gcctgggtgatgagaaataat-3′	109 bp
		Reverse	5′-gctgacctgtgcctgtaaaa-3′	
		Probe	FAM 5′-acactgacccatgaagagaaagcagt-3′BHQ1	
4q22.1	ABCG2 NG_032067.2	Forward	5′-gagttggtttgtgcttgtgtt-3′	100 bp
		Reverse	5′-attccattttaagtcaggttctatt-3′	
		Probe	FAM 5′-agggtaggcactgaatatactcaatga-3′BHQ1	
16p11.2	MVP NC_000016.10	Forward	5′-cctgaaacagcacaggactg-3′	
		Reverse	5′-tggaagcacccgcaaccctaa-3′	
		Probe	FAM 5′-agggtaggcactgaatatactcaatga-3′BHQ1	
3q13.33	*CASR* NG_009058.1	Forward	5′-ccacctccacaacagcct-3′	105 bp
		Reverse	5′-gctggaggaggcataactga-3′	
		Probe	FAM 5′-ctcagcacctcttcactcactcact-3′BHQ1	

NG – number according to NCBI Nucleotide Database (http://www.ncbi.nlm.nih.gov/nuccore); bp: base pair; Forward – forward primer; Reverse - reverse primer.

### Statistical analysis

The presence of structural CNV in MDR gene loci was assessed using the Chromosome Analysis Suite 2.0 software. Statistical analyses were performed using the STATISTICA 8.0 software (StatSoft, Tulsa, OK, USA). The arithmetic mean value and standard error were calculated for each sample group, and Mann-Whitney *U*-test was applied to identify the link between the expression levels of MDR genes in breast tumors and the presence/absence of deletion in the MDR loci. A two-sided *p*-value was calculated using Fisher's exact test http://vassarstats.net/odds2x2.html, and the Bonferroni correction was applied to address the problem of multiple comparisons and was calculated as the each *p*-value multiplied by the number of comparisons [46].
